# WAIST-TO-HEIGHT RATIO INDEX OR THE PREDICTION OF OVERWEIGHT IN CHILDREN

**DOI:** 10.1590/1984-0462/;2018;36;1;00002

**Published:** 2017-11-17

**Authors:** Sarah Aparecida Vieira, Andréia Queiroz Ribeiro, Helen Hermana Miranda Hermsdorff, Patrícia Feliciano Pereira, Silvia Eloiza Priore, Sylvia do Carmo Castro Franceschini

**Affiliations:** a Universidade Federal de Viçosa, Viçosa, MG, Brasil.

**Keywords:** Child, Overweight, Obesity, Abdominal obesity, ROC curve, Criança, Sobrepeso, Obesidade, Obesidade abdominal, Curva ROC

## Abstract

**Objective:**

To identify a low-cost abdominal adiposity index that has a higher accuracy in predicting excess weight in children aged four to seven years old.

**Methods:**

A cross-sectional study with a sample of 257 children aged 4 to 7 years old. Indicators of abdominal adiposity assessed were: waist circumference (WC), waist-to-height ratio (WHR) and central fat percentage (measured by dual energy X-ray absorptiometry - DEXA). Overweight children were classified using body mass index by age (BMI/age). In the analysis, the prevalence ratio (PR) using Poisson regression with a robust variance was estimated, and a receiver operating characteristic (ROC) curve was built, with a statistical significance of p<0.05.

**Results:**

The prevalence of overweight children was 24.9% and a higher median of all abdominal adiposity indicators was observed in the overweight group. Children with increased values of WC (PR=4.1; 95%CI 2.86-5.86), WHR (PR=5.76; 95%CI 4.14-8.02) and a central fat percentage (PR=2.48; 95%CI 1.65-3.73) had a higher prevalence of being overweight. Using the ROC curve analysis, the WHR index showed a higher area under the curve, when compared to the WC and to the central fat percentage estimated by DEXA for predicting the classification of being overweight.

**Conclusions:**

Given the results, WHR is suggested for the screening of overweight children.

## RESUMO

**Objetivo:**

Identificar um indicador de adiposidade abdominal de baixo custo e com maior acurácia para predizer o excesso de peso em crianças de quatro a sete anos idade.

**Métodos:**

Estudo transversal com amostra de 257 crianças de 4 a 7 anos. Os indicadores de adiposidade abdominal avaliados foram: perímetro da cintura (PC), relação cintura-estatura (RCE) e percentual de gordura central (avaliado pela técnica *dual energy X-ray absorptiometry* - DEXA). O excesso de peso foi classificado pelo índice de massa corporal por idade (IMC/I). Nas análises, estimou-se a razão de prevalência (RP) pela regressão de Poisson com variância robusta e utilizou-se a curva (*receiver operating characteristics* ­- ROC), considerando como significância estatística p<0,05.

**Resultados:**

A prevalência de excesso de peso foi de 24,9%, e observou-se maior mediana dos indicadores de adiposidade abdominal no grupo de crianças com excesso de peso. As crianças com valores aumentados de PC (RP=4,1; IC95% 2,86-5,86), RCE (RP=5,76; IC95% 4,14-8,02) e percentual de gordura central (RP=2,48; IC95% 1,65-3,73) apresentaram maior prevalência de excesso de peso. Verificou-se, na análise de curva ROC, que o índice RCE apresentou maior área sob a curva, comparado ao PC e ao percentual de gordura central estimada pelo DEXA, na predição do excesso de peso.

**Conclusões:**

Diante dos resultados, sugere-se a utilização da RCE para triagem de crianças com excesso de peso.

## INTRODUCTION

High rates of overweight and obese children point to a serious public health issue.^1^ In addition, the prevalence of morbidities associated with being overweight, such as dyslipidemias, type 2 diabetes, hypertension and metabolic syndrome, and psychological problems like depression and low self-esteem, are increasing.^2^ ^,^ ^3^

A global estimate published in 2010 showed that 35 million children worldwide were overweight or obese, and this figure is expected to double by 2020.^4^ In Brazil, according to data from the Family Budget Survey (*Pesquisa de Orçamentos Familiares* - POF), carried out in 2008 and 2009, 35% of children aged 5 to 9 years are overweight; 16.6% of boys are obese, and among girls, obesity reached 11.8%.^5^

Epidemiological and clinical studies have shown that, regardless of being overweight, the location and distribution of body fat are associated with cardiometabolic risk factors in the early stages of life, such as childhood and adolescence.^6^ ^,^ ^7^ Therefore, the identification of simple and accurate methods that assess body adiposity in children is key for clinical practice.

There are different methods for assessing body composition. Magnetic resonance and computed tomography are considered the most accurate because they allow for a differentiation between subcutaneous fat and visceral fat. However, they are not common in clinical practice or in research because of their high cost and the child’s exposure to ionizing radiation, in the case of tomography.^8^ The dual energy X-ray absorptiometry (DEXA) technique shows good accuracy with low radiation levels and is therefore indicated to evaluate body composition in children.^9^

Body Mass Index (BMI) is recommended for assessing the nutritional status of children and is widely used with pre-established cutoff points.^3^ ^,^ ^10^ ^,^ ^11^ However, this index has some limitations, such as the lack of differentiation between subcutaneous fat, visceral fat, and muscle mass of the adipose. Thus, other measures and indices such as waist circumference (WC) and waist-to-height ratio (WHtR) have been increasingly used to assess the location of body fat, but little is known about their capacity to predict excess body weight in children.^12^ ^,^ ^13^ ^,^ ^14^ These are easy, innocuous and inexpensive measures, which have been associated with cardiometabolic risk in studies.^7^ ^,^ ^12^ ^,^ ^14^

In view of the above, this study aimed to identify a low-cost indicator of abdominal adiposity with the greatest accuracy to predict overweight children aged four to seven years old.

## METHOD

A cross-sectional study was carried out with children aged four to seven years old, who were born in the only maternity hospital in Viçosa, Minas Gerais, Brazil, and who were monitored by the Lactation Support Program (*Programa de Apoio à Lactação* - PROLAC) in the first six months of life. PROLAC is an Extension Program of the Universidade Federal de Viçosa (UFV), in partnership with the municipality’s Human Milk Bank, which began in 2003. Among its activities, PROLAC provides guidance to mothers in the postpartum period. It aims at promoting breastfeeding and nutritional care for mothers and their infants in their first year of life.

The children were recruited based on the selection of PROLAC care records, and two inclusion criteria were adopted: the presence of identification data that showed the location of the children, and that the child’s date of birth was compatible with the ages between four and seven years old at the time of study. Of the 371 children eligible to participate, 78 were not located (change of address) after at least three home visit attempts; 29 were not authorized by parents to participate or did not complete all stages of the study; and 7 had health problems that prevented their participation. Thus, 114 losses were recorded (30.7%) and the sample of the study was 257 children.

After data collection, the power of the study was calculated, considering the outcome of the WC measurement between the two nutritional status groups (eutrophic and overweight). Based on the means and standard deviations of the WC in the group of eutrophic (51.6±3.2 cm) and overweight children (60.5±5.7 cm), the sample calculation indicated that evaluating 193 eutrophic children and 64 overweight children had a power equal to 100% at a significance level of 5%. The OpenEpi software (www.OpenEpi.com) was used for this analysis.

Weight was obtained using an electronic digital scale with a capacity of 150 kg and an accuracy of 10g. Height was measured using a vertical stadiometer mounted on the wall, with a length of 2 m, divided in centimeters and subdivided in millimeters. Being overweight was classified by BMI/age (BMI/A) according to gender, using the z-score +1 from the World Health Organization (WHO) as the cut-off point.^10^

To measure the WC, a flexible and inelastic measuring tape of 2 m that was divided into centimeters and subdivided into millimeters, was used at the level of the umbilical scar. The measurements were performed three times, and the two closest ones were calculated to determine the mean.

An evaluation of body composition was performed using the DEXA technique, and the fat percentage in the central region was adopted for analysis. The 75^th^ percentile of the sample, according to sex and age, was considered for the classification of WC values and percentage of fat in the central region.^15^ The WHtR was calculated by the ratio of waist circumference (cm) and height (cm), considering risk values of ³0.5.^16^

A semi-structured questionnaire was applied to obtain sociodemographic (maternal schooling) and lifestyle (daily time watching television and sports practice) information. The child’s guardian was asked to fill out three dietary records for the child on non-consecutive days, including one on the weekend. The analysis of dietary records was performed using Dietpro software, version 5i. In order to determine energy balance, the estimated energy requirement (EER) was calculated and compared to the average daily caloric intake, obtained by an analysis of the records.^17^

In the statistical analysis, the distribution of variables was initially verified by the Kolmogorov-Smirnov normality test. The descriptive analysis of the data was performed by central tendency and dispersion measures. The Mann-Whitney test was applied to identify the statistical difference of the study variables between the two nutritional status groups.

In the bivariate analysis, the prevalence ratio (PR) and the 95% confidence interval (95%CI) were estimated using Poisson regression, and the variables that showed p<0.20 were considered for inclusion in the multiple model with robust variance. The receiver operating characteristic (ROC) curve was used to assess the accuracy of abdominal adiposity indicators in the prediction of excess weight. The analyses were performed using Stata software version 13.0 (Stata Incorporation, Texas, USA) and Statistical Package for Social Science (SPSS) version 21 (SPSS Incorporation, Chicago, USA). The statistical significance considered was p<0.05.

This study was approved by the Ethics Committee in Research with Human Beings of the Universidade Federal de Viçosa (Protocol No. 094/2011). Participation was voluntary and the children’s guardians signed an Informed Consent form.

## RESULTS

The study sample consisted of 257 children; 55.2% were males, and the median age was 73 months (6 years) old. The prevalence of excess and low weight was 24.9 and 2.7%, respectively. The highest median of all abdominal adiposity indicators evaluated was observed in the group of overweight children, for both sexes (Table 1).

**Table 1:** Abdominal adiposity indicators in children aged four to seven years old, according to nutritional status and sex.

	Not overweight (n=193)			Overweight (n=64)			
	Med	Min	Max	Med	Min	Max	p-value*
Boys							
WC (cm)	52.20	42.20	59.50	59.40	51.80	80.10	<0.001
WHtR	0.45	0.30	0.50	0.49	0.40	0.60	<0.001
Central fat (%)	6.00	4.00	27.40	16.90	4.20	41.90	<0.001
Girls							
WC (cm)	51.10	44.30	50.20	60.50	46.30	68.80	<0.001
WHtR	0.44	0.30	0.50	0.48	0.40	0.50	<0.001
Central fat (%)	9.20	4.00	31.40	24.90	11.10	4.40	<0.001

WC: waist circumference; WHtR: waist-to-height ratio; cm: centimeter; med: median; min: minimum; max: maximum; *Mann-Whitney test.

It was observed that being overweight was more prevalent among children who presented WC values and percentage of central fat ³ to the 75^th^ percentile and WHtR³0.5. In addition, girls were more protected against being overweight. The other sociodemographic and lifestyle variables evaluated did not differ significantly between the group that was overweight and the group that was not (Table 2).

**Table 2:** The prevalence of overweight children and crude prevalence ratios, according to abdominal adiposity indicators, sociodemographic and lifestyle variables in children aged four to seven years old.

	Not overweight n (%)	Overweight n (%)	PR (95%IC)
WC			
<p75	182 (82.7)	38 (17.3)	1
≥p75	11 (29.7)	26 (70.3)	4.06 (2.84-5.81)*
WHtR			
<0.5	189 (84.8)	34 (15.3)	1
≥0.5	4 (12.5)	28 (87.5)	5.73 (4.09-8.03)*
Central fat %			
<p75	175 (79.6)	45 (20.5)	1
≥p75	18 (48.7)	19 (51.4)	2.51 (1.66-3.77)*
Sex			
Male	98 (69.0)	44 (31.0)	1
Female	95 (82.6)	20 (17.4)	0.56 (0.33-0.95)**
Age (years)			
4-5	95 (79.8)	24 (20.2)	1
6-7	98 (71.0)	40 (29.0)	1.43 (0.92-2.23)
Maternal schooling (years)			
>8	110 (73.3)	40 (26.7)	1
≤8	81 (77.1)	24 (22.9)	0.85 (0.55-1.33)
Sports practice			
Yes	13 (61.9)	8 (38.1)	1
No	180 (76.3)	56 (23.7)	0.62 (0.34-1.12)
Daily time watching TV (hours)			
≤2	90 (79.7)	23 (20.4)	1
>2	103 (71.5)	41 (28.6)	1.39 (0.89-2.18)
Energetic balance			
Not positive	143 (73.7)	51 (26.3)	1
Positive	50 (79.4)	13 (20.6)	0.78 (0.45-1.34)

P: percentile; WC: waist circumference; WHtR: weight-to-height ratio; PR: prevalence ratio; 95%IC: 95% confidence interval; *p<0.001; **p<0.05.

After adjustment for gender, age, sports practice and daily time in front of the television, all of the abdominal adiposity indicators analyzed were associated with being overweight. Children with an increased WC and percentage of central fat showed, respectively, 4.1 and 2.5 times the prevalence of excess weight compared to those with a normal weight. Regarding the WHtR index, the prevalence was 5.8 times higher in the group with WHtR³0.5 (Tabela 3).

**Table 3:** Final model of the Poisson regression analysis for the abdominal adiposity variables associated with overweight children aged four to seven years old.

	Adjusted PR	95%CI	p-value*
WC^a^			
≥p75	4.10	2.86-5.86	<0.001
WHtR ^a^			
≥0.5	5.76	4.14-8.02	<0.001
Central fat%^a^			
≥p75	2.48	1.65-3.73	<0.001

P: percentile; WC: waist circumference; WHtR: weight-to-height ratio; PR: prevalence ratio; 95%CI: 95% confidence interval; ^a^adjusted by sex, age, sports practice and daily time watching TV; *multiple Poisson regression with robust variance.

**Figure 1:**
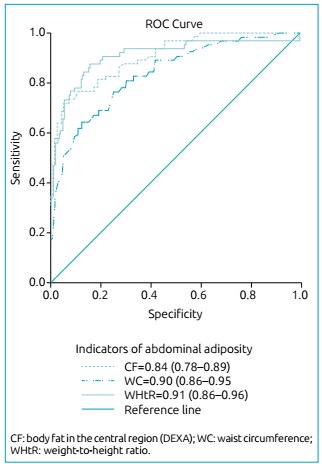
ROC curve of abdominal adiposity indicators used as predictors of overweight children aged four to seven years old.

The ROC curve (Figure 1) shows that, among the indicators of abdominal adiposity, the WHtR (area under the curve - AUC=0.91; 95%CI 0.86-0.96) showed the greatest accuracy in the prediction of excess weight among children, followed by WC (AUC=0.90; 95%CI 0.86-0.95) and central fat percentage estimated by DEXA (AUC=0.84; 95%CI 0.78-0.89).

## DISCUSSION

The WHtR was the abdominal adiposity indicator that presented the greatest area under the curve in the prediction of excess weight in children aged four to seven years old, despite the overlap of confidence intervals. All of the indicators evaluated (WC, WHtR and percentage of central fat) showed a higher median in the group of overweight children, and this result is in agreement with that observed in other studies that evaluated children and adolescents.^7^ ^,^ ^12^

The logical basis of the WHtR is that for a given height, there is an acceptable degree of fat stored in the trunk region.^18^ In the present study, it was observed that children with a WHtR³0.5 value presented 5.6 times a greater risk of being overweight, compared to children with an WHtR<0.5. A similar result was observed in a study conducted in southern Brazil with children aged six to ten years, in which it was suggested that the WHtR could be used as a complementary parameter to BMI/A to determine abdominal adiposity in that population.^19^ Although other cutoff points of WHtR (mostly <0.5) have been suggested to assess abdominal adiposity, the cutoff point of 0.5 was established as appropriate in several studies with children.^13^ ^,^ ^20^ Furthermore, because it is a single cutoff point that is applicable for both sexes and all age groups, regardless of ethnicity, it makes its application and the interpretation of its results easier.^16^

It was observed that the WHtR was the best predictor to classify the nutritional status of the children evaluated (AUC=0.91; 95%CI 0.86-0.96). The WC measure alone showed a high area value under the curve (AUC=0.90; 95%IC 0.86-0.95), and was very close to that found for the WHtR. In the study carried out by Brambilla et al.,^11^ with children and adolescents aged 8 to 18 years, it was also observed that the WHtR, compared to the WC, was the best predictor of abdominal adiposity in the population evaluated. A controversial result was observed in a study conducted with Venezuelan children and adolescents between the ages of 7 and 17, in which authors concluded that the WHtR did not effectively identify the distribution of body fat due to the low values of sensitivity and specificity.^21^

The evaluation of the nutritional status of children using BMI/A showed a prevalence of low weight in 2.7% of them, and of excess weight in 24.9%, demonstrating the process of nutritional transition that has been occurring in Brazil. It is characterized by the reduction of weight deficit and increase in excess weight.^5^ In the study by Gigante et al.^22^ in Pelotas, Rio Grande do Sul, birth cohorts of 1982 and 1993 were compared, showing an approximately twofold increase in the prevalence of overweight children born in 1993, when compared, in a similar age, to those born in 1982. On the other hand, there was a decrease of almost 50% in the prevalence of short stature, when the same children were compared in both periods. This tendency was also verified in the results of the last Family Budget Survey,^5^ characterizing the process of nutritional transition.

BMI has been used in studies to assess nutritional status due to its correlation with total and visceral body fat, which is considered an important risk factor for chronic-degenerative diseases.^3^ ^,^ ^23^ The relationship between such morbidities and BMI is already well known in adults; however, in children, it becomes more difficult to establish, since such changes commonly manifest themselves in later stages of life.

In the present study, it was observed that children with a WC ³ the 75^th^ percentile were 4.1 times more likely to be overweight, compared to those with lower percentiles, even after adjusting for other variables. This result is in agreement with other studies, which found a strong correlation between BMI and WC.^19^ ^,^ ^24^ When carrying out a cross-sectional study with preschool children of low socioeconomic status, Sarni et al.^23^ also observed a strong correlation between these two parameters in the evaluation of abdominal adiposity (r=0.87; p<0.001). In a study of 2,597 children and adolescents aged 5 to 18 years that belonged to the Bogalusa Heart Study, there was a strong correlation between BMI and WC (r=0.92; p<0.001). The authors concluded that, despite the strong correlation between the two indicators, the combined use of the two markers proved to be a good predictor of health risks in the pediatric population.^25^

WC is the most widely used measure in the assessment of abdominal adiposity, and many authors address the ability of this indicator to evaluate abdominal fat in children.^7^ ^,^ ^12^ ^,^ ^23^ ^,^ ^26^ However, there are different anatomical sites to measure WC, which makes it difficult to compare the results of the studies. In the study by Bosy-Westphal et al.,^27^ carried out with children, it was observed that the WC values differed among the evaluation sites. In a study conducted with 205 children aged 6 to 9 years old, it was observed that the waist measurement performed at the midpoint between the iliac crest and the last rib with the percentage of body fat evaluated using the four-pole bioimpedance, showed a higher correlation (r=0.50 in boys; r=0.62 in girls) compared to the measurement performed on the lower perimeter of the abdomen (r=0.49 in boys; r= 0.59 in girls).^28^

In relation to the percentage of body fat evaluated by DEXA, after adjusting for other variables, the present study found that children with increased central fat percentage presented a 2.48 times greater prevalence of being overweight, compared to those with a lower percentage of fat in that area. There are few studies in the literature that evaluated the percentage of abdominal body fat in children.^27^ ^,^ ^29^ Those which estimated the percentage of total body fat, without discriminating based on the area, were the most common.^14^ ^,^ ^28^

There is not yet a consensus in the literature about which cutoff points for WC and percentage of body fat would be adequate to classify these parameters in children, which makes it difficult to compare the results of the studies.^15^ ^,^ ^30^ Research involving diagnostic tests, such as sensitivity and specificity, is necessary for the definition of appropriate cutoff points for these indicators in children.

As presented in Table 2, when a group of eutrophic children was evaluated by means of abdominal adiposity indicators, the prevalence of altered nutritional status was higher when compared to the BMI/A classification. In addition, the WHtR was the indicator that showed the greatest potential for evaluating overweight children, and 87.5% of the children with this condition had a value for WHtR³0.5. Additionally, the WHtR index has other advantages when compared to several methods, such as being low cost, easily obtained (only height and WC measurements are necessary), easily interpreted, and useful for various health professionals.

The main limitation for this study was the fact that it was not a population-based survey, with a representative sample. Therefore, the observed results should be extrapolated with caution to other populations. However, the conclusions obtained can be used as a starting point for future studies. It is worth highlighting, as a positive point of this study, the inclusion of potential confounding variables in the analyses, which may influence nutritional status and body composition in childhood. Thus, it was possible to evaluate independently the association of abdominal adiposity indicators with excess weight among the evaluated children.

It can be concluded that, for both genders, all of the abdominal adiposity indicators evaluated presented a higher median in the group of overweight children. In addition, the prevalence of changes in these indicators was higher in this group, after adjusting for socioeconomic and lifestyle variables. Children with increased abdominal adiposity had a higher risk of being overweight, considering the three indicators evaluated. The WHtR was the measure that had the greatest accuracy in the prediction of overweight children in the study, emphasizing its use in screening children with excess weight and abdominal adiposity.

Considering that excess abdominal fat represents a risk factor for cardiometabolic diseases, the use of indicators to assess adiposity from childhood, such as WHtR, is recommended. We still need population-based studies, with representative samples, that seek to propose cutoff points for the classification of abdominal adiposity in children.
